# Development of the Cardiovascular Assessment Screening Program (CASP) using the qualitative findings of a mixed methods study and applying the TDF to address the barriers of and facilitators to comprehensive screening for cardiovascular disease

**DOI:** 10.1186/s12875-023-02022-8

**Published:** 2023-03-07

**Authors:** Jill Bruneau, Karen Parsons, Donna Moralejo, Catherine Donovan

**Affiliations:** 1 Faculty of Nursing, Memorial University of Newfoundland, 323 Prince Philip Drive, St. John’s, NL A1B 3X8 Canada; 2 Faculty of Medicine, Memorial University of Newfoundland, 300 Prince Philip Drive, St. John’s, NL A1B 3V6 Canada

**Keywords:** Clinical practice guidelines, Theoretical Domains Framework, Cardiovascular, Screening, Intervention development, Mixed methods

## Abstract

**Background:**

There is inconsistent utilisation of clinical practice guidelines (CPGs) for cardiovascular disease (CVD) screening and management by healthcare professionals to identify CVD risk factors early and to intervene using current recommendations. This manuscript reports on the first phase of an exploratory sequential mixed methods study describing the integration of the qualitative study findings with the Theoretical Domains Framework (TDF) that led to the development of the Cardiovascular Assessment Screening Program (CASP). The main objective of the qualitative study was to inform the development of CASP.

**Methods:**

Focus groups (5) and interviews (10) were conducted in rural and urban settings in one Canadian province with target health professionals, managers in health care organizations, and the public to obtain different perspectives to inform the CASP intervention. Three focus groups were held with nurse practitioners and two with members of the public; individual interviews were conducted with target groups as well. Application of the TDF provided a comprehensive approach to determine the main factors influencing clinician behaviour, to assess the implementation process, and to support intervention design. Behaviour change techniques, modes of delivery, and intervention components were selected for the development of the CASP.

**Results:**

Themes identified such lack of knowledge about comprehensive screening, ambiguity around responsibility for screening, lack of time and commitment to screening were addressed in the components of the CASP intervention that were developed, including a website, education module, decision tools, and a toolkit.

**Conclusion:**

CASP is a theory-informed intervention developed through the integration of the findings from the focus groups and interviews with selected TDF domains, behaviour change techniques, and modes of delivery available in the local context that may be a useful approach for knowledge translation of evidence into practice.

**Supplementary Information:**

The online version contains supplementary material available at 10.1186/s12875-023-02022-8.

## Contributions to the literature

- Illustrates a systematic process of integrating qualitative research findings and knowledge translation theories to develop an intervention.
- The comprehensive systematic approach used in the CASP intervention helps to translate the C-CHANGE guideline for use by healthcare providers so that they can identify and manage multiple risk factors simultaneously, rather than focusing on only a few risk factors as often occurs in screening interventions.
- The CASP intervention, based on current clinical practice guidelines C-CHANGE, addresses the barriers and facilitators of screening for cardiovascular disease and promotes comprehensive systematic screening by health providers in clinical practice.

## Background

The uptake and utilisation of clinical practice guidelines (CPGs) by healthcare providers (HCPs), such as family physicians and nurse practitioners (NPs), occur inconsistently for the screening, diagnosis, and management of many chronic conditions, including cardiovascular disease (CVD) [1–3]. CVD is the number one cause of death globally and accounts for approximately 30% of total deaths in Canada [4, 5]. CVD morbidity results in lost years of life, reduced productivity, and decreased quality of life for many individuals and families [6]. CVD includes diseases of the heart, vascular diseases of the brain, and diseases of the blood vessels that can progress to myocardial infarctions and cerebrovascular accidents leading to increased morbidity and mortality [5]. Utilisation of CPGs by health professionals for the screening, diagnosis, and management of CVD is quite complex due to the many factors and multi-morbidity that influence CVD development [1–3]. For example, diabetes, dyslipidemia, and hypertension are comorbid conditions that contribute to the development of CVD. Each risk factor or condition has different CPGs that often overlap, making it difficult for clinicians to stay abreast of the most current research in order to individualise care for people at high risk for developing CVD.

There is evidence that screening to identify cardiovascular risk factors and conditions early, can reduce morbidity and premature mortality [7, 8]. Individualised approaches for behaviour change instituted by HCPs, with attention to socioenvironmental influences that affect daily life, may reduce morbidity and premature morality from CVD.

Because of this and other barriers such as lack of time and resources, there are inconsistencies in the utilisation of the cardiovascular CPGs by HCPs [1, 3]. Current recommendations supporting CVD screening and management in Canada are in a set of coordinated CPGs called the Canadian Cardiovascular Harmonized and National Guideline Endeavour known as C-CHANGE [9]. The C-CHANGE guideline is comprehensive and multifaceted addressing many different risk factors and conditions. Even though the C-CHANGE guideline is published in an online journal, the daily application of this guideline is difficult for clinicians because the screening and the management recommendations for many chronic conditions are presented together and this complex nature of the guideline make it burdensome for deciding on appropriate actions during patient encounters.

To address this clinical practice issue and to increase utilisation of the C-CHANGE guideline by HCPs such as NPs, we conducted a multiphase exploratory sequential mixed method study to determine successful strategies for implementation of CPGs through the development, implementation, and evaluation of a cardiovascular screening intervention. To ensure that the intervention was contextually and culturally relevant, we used the planned action framework, Knowledge to Action (KTA) cycle, with guideline adaption [10]. We used this framework to guide our study and embedded the C-CHANGE guideline into the CASP intervention. Following the planned action framework, KTA cycle, the first step was to choose the knowledge to translate that, in this case, was the C-CHANGE guideline. The second step was to adapt the knowledge to the local context. Researchers conducted focus groups and interviews with HCPs, managers of health organisations, and the public in various areas of the province to gain different perspectives to develop a tailored intervention that was relevant to our context prior to implementation. Researchers utilised a mixed methods study design to ensure that the findings from the qualitative phase could be integrated using the Theoretical Domains Framework (TDF) to develop a novel intervention to be later tested in the second phase of the study. The integration phase of the exploratory mixed methods study involved using the qualitative findings (themes) to build a new intervention called Cardiovascular Assessment Screening Program (CASP) within which the C-CHANGE guideline was embedded. In this building process, the researchers had to decide/make key decisions about what aspects of the qualitative findings to build on and the nature of the quantitative entity to be built [11]. The objective of the qualitative study was to inform the development of CASP. In our research, the TDF (that focuses on behaviour change of NPs in uptake of CPGs) was used to determine what barriers, facilitators, and strategies would need to be addressed in the development of the CASP intervention [12].

We chose the TDF for this study to provide a comprehensive approach to determining the main factors influencing clinician behaviour, to assess the implementation process, and to support intervention design [12–14]. While the identification of barriers and facilitators to CVD screening using CPGs by HCPs were the main findings of the qualitative study that informed the development of the intervention, utilising the TDF as our theoretical foundation for developing our intervention was an important part of the research process. Originally, the TDF was developed by identifying theories relevant to implementation and grouping the constructs from these theories into domains [13]. The TDF contains a set of 14 domains covering the main factors influencing practitioner clinical behaviour [14–16]. For the purpose of our mixed methods study, we selected specific domains from the TDF that matched phase 1 qualitative study findings, then in the integration phase being discussed in this article, we selected then developed the implementation intervention designed to change clinician behaviour and improve the uptake of C-CHANGE in daily clinical practice. Utilising a theoretical basis with its focus on changing clinician behaviour was critically important, rather than randomly selecting the components of the intervention, in order to promote changes in behaviour and to increase the likelihood of successful intervention implementation by HCPs.

Once the CASP intervention was developed, the next step of the KTA framework was to test it. In phase 2 of the study, we tested this evidence-informed intervention with NPs in community clinics in one Canadian province, Newfoundland and Labrador (NL) [17]. The NPs collaborated with their patients to test the effectiveness of the CASP intervention using a small cluster randomized controlled trial (cRCT) in community practice settings across NL. At the end of phase 2, there was a separate integration phase when the effectiveness findings were integrated with the qualitative findings to refine the CASP intervention [17].

### Purpose

The purpose of this paper is to present the integration of the qualitative study findings with the TDF that led to the development of the CASP intervention. This implementation intervention operationalised the C-CHANGE guideline and made it more user-friendly for HCPs to screen comprehensively in daily practice. We followed a process similar to others who developed interventions to promote adherence to guidelines [12, 18]. In previous studies, researchers have applied the TDF and determined the barriers and facilitators to clinician behaviour change related to the adherence to national guidelines [12, 18]. Many studies have described the barriers and facilitators of recommended practice and have utilised the TDF as a guide to develop interventions aimed at translating evidence from clinical guidelines into practice [18]. Other studies have described the matching of the “domains” of the TDF with behaviour change techniques [13, 15]. There have also been studies that have described the specific modes of delivery that are relevant to clinical practice [12, 18]. Our research study adds to the body of knowledge through its application of the TDF as a framework, use of the behaviour change taxonomy for BCTs, and modes of delivery relevant to the local context for the CASP intervention development.

There were five steps in the process to develop the theory-informed intervention, summarised in Table 1. The first step was to identify the guidelines to be implemented; the remaining steps are discussed in detail in this paper. Researchers used recommended reporting guidelines, namely, the Standards for Reporting Qualitative Research (SRQR) checklist [19].

**Table 1:** Overview of the Process for the Development of a Theory-informed Intervention, CASP

**Steps**	**Actions**
**1. Identify the target behaviour of the HCP inconsistent use of CPGs in daily clinical practice for comprehensive CVD screening and management.**	1. Performed a literature review to identify the target behaviour and to find effective interventions to promote HCP adherence to the C-CHANGE guideline.
**2. Explore the barriers and facilitators related to CVD screening and find possible strategies within the local context.**	2. Conducted focus groups and individual interviews using evidence-informed interview guides with HCPs, managers and interested members of the public; research team consensus on findings.
**3. Match barriers and facilitators with potential solutions for clinician behaviour change during the integration of phase 1 findings.**	3. Identified barriers and facilitators from research findings to: (a) match with the theoretical domains of the TDF and (b) choose relevant behaviour change techniques; research team discussion.
**4. Combine the behaviour change techniques with the modes of delivery and strategies for the intervention.**	4. Selected the modes of delivery congruent with the local context; researchers and technical support available at the local university to support the intervention components; research team consensus on intervention.
**5. Finalize the CASP intervention components.**	5. Obtained feedback on CASP from knowledge users and patient partners; reviewed components of intervention with research team.

## Methods

### Qualitative approach and research paradigm

The methodological philosophy and qualitative approach of interpretive description (ID) was used in this qualitative study [20]. ID enables researchers of various disciplines to utilise applied qualitative research in a pragmatic way to address real-life issues or problems identified in the field and to find solutions that could be useful in the practice setting. Our research study addressed the issue of lack of uptake and utilisation of CPGs by HCPs to promote knowledge translation of evidence into daily practice.

### Trustworthiness

Trustworthiness as it pertains to qualitative research was an important consideration in this research. Trustworthiness of the research was accomplished in two ways, by reflexivity [21, 22] and triangulation [23, 24]. The principal investigator (PI) was an NP with clinical experience and expert knowledge. The PI had a collegial relationship with some of NP participants or had previously taught some of the practicing NPs. The PI also knew a number of patient participants and two members of the public. Having former relationships with participants had the potential to influence the data analysis and dissemination. Because the PI was aware of this potential influence, she purposely attempted not to be unduly swayed by previous relationships during the analysis and interpretation of the data. For example, when conducting interviews the PI was careful not to use probing questions, facial expression or body language so to not influence what the participant communicated. Another way trustworthiness of the research was accomplished was by researcher triangulation [23]. At least one other researcher crosschecked the PI’s work by following the decision-trail of the PI, and provided feedback about the analysis and results. To strengthen credibility, we conducted peer debriefing and reviewed the data, codes, and themes. During data analysis and interpreting data, the PI positioned herself as an “insider” during the research process so to be open to new ideas and knowledge from the participants in order to gain new knowledge related to the realities of these participants during data collection, and analyses. Multiple realities existed from the various participants. Continuous self-reflection occurred so that PI’s knowledge and reality was at the forefront and acknowledged during the research process. Reflective journaling was also important in order to minimise bias as much as possible. The researchers, familiar with the ID methodology and constant comparative methods, ensured that any conclusions drawn were truthful representations of the participants’ experiences.

### Context

As per Step 2 in Table 1, we conducted focus groups and individual interviews with HCPs, managers, and the public to inform the development of the CASP intervention. These interviews and focus groups occurred in various locations across the province of NL, Canada, in both rural and urban settings within two of the four regional health authorities (RHAs) in the province.

### Research questions

We sought to answer two research questions: 1) What are the facilitators and barriers associated with CVD screening of at-risk individuals aged 40–74 years in NL, Canada?, and 2) What are the tools and strategies that various health care providers, health managers, and members of the public recommend to increase comprehensive CVD screening in NL, Canada?

### Sampling strategy

The target groups for this study were nurse practitioners, nurses, family physicians, dietitians and pharmacists, healthcare managers, and members of the public. A convenience sample representing various members of the interprofessional team, healthcare managers, and members of the public was recruited from both urban and rural areas in NL. We thought it important to include individuals representing different professional groups having varied experiences with CVD screening and management and with different perspectives to provide unique contributions to inform the development of the intervention. Engaging knowledge users and patient partners as collaborators in a patient-centred care approach for the intervention during the design phase of intervention was done to ensure that the end program or intervention was relevant to the context and potentially sustainable [25].

There were 30 participants involved in phase 1 of this study. This number was thought to be a sufficient sample and was consistent with ID methodology [20]. We recruited participants (*n*= 30) from three different target groups: 1) health professionals (nursing, pharmacy, dietetics, and medicine), 2) managers and 3) the public (See Additional file 1 Phase 1 Participants). We used three different interview guides for the target groups. We recruited as many participants as we were able to recruit for each group to gain a variety of perspectives on the barriers and facilitators for CVD screening. There was no set cut-off of the number of participants from each of the target groups. Of note, researchers did experience significant difficulty in the recruitment of busy health professionals for both phases of the study as described elsewhere [26].

### Data collection methods

Data collection began once we received approval for our study from the Human Research Ethics Board (HREB) at Memorial University of Newfoundland. All interviews and focus groups were digitally recorded, field notes were taken, and were recorded as personal responses to what had been learned or observed during the focus groups sessions. All focus groups and interviews were transcribed verbatim and field notes further guided the content and interpretation of the data. Focus groups enabled us to obtain enriched responses as a result of group dynamics and allowed for reflection and expression of ideas by group members of the same professional group that may not otherwise be revealed in an individual interview [27]. Individual interviews, either face-to face or telephone, were conducted for those individuals who were unable to participate in a focus group. Each focus group lasted 60–90 min and the individual interviews were approximately 60 min. We developed three different semi-structured interview guides, informed by the literature, used with the different participant groups (HCPs, healthcare managers and the public) (see Additional file 2) for the semi-structured interview guides [3, 9, 28–32]. Interview guides used during the focus groups and individual interviews were adapted according to the target group over the duration of the data collection period.

#### Data analysis

Data were analyzed using constant comparative strategy as described by Kruger and Casey [27]. All data were uploaded into NVIVO software for data management and coding [33]. One study investigator (JB) coded all of the transcripts line by line and further organized into nodes in NVIVO according to the semi-structured interview guide questions. Codes, categories and patterns were developed using a systematic approach. This involved the researchers (JB and KP) independently looking at each research question, identifying data in relation to this question, breaking down the textual data, and then collecting it together into categories and patterns respectively on the grounds that it shares similar characteristics in relation to the given research question. This process was repeated for each question. Then, together, the investigators JB and KP compared patterns within and across all groups to construct the main ideas. In the event of no agreement between the researchers, a third researcher would be consulted. This was not necessary, as JB and KP were able to reach agreement [27]. The principles of content analysis were used to classify the data according to themes; this facilitated the application of narrative analysis principles. The following issues were also considered: how data linked together, the purpose behind the data, and key messages in the text and their functions. A code system was developed based on the meanings the data suggested. In keeping with ID [20], these patterns were further integrated and synthesized, to inform the development of relevant content and implementation strategies for CASP. Techniques to enhance credibility of the research findings were used consistent with ID methodology in terms of purpose, process, and context. ID encourages obtaining a variety of different perspectives from participants. We were interested in different perspectives on the barriers and facilitators of CVD screening from not only the perspective of HCPs but also managers in organizations and the recipients of such a screening program, the patients or members of the public. We thought it was important to gain a broader understanding of the idea of systematic CVD screening from a variety of participants knowing that there would always be more to learn. We did document commonalities in the responses to the interview questions from the NP interviews and focus groups using the constant comparative method, however, due to the limited number of participants from pharmacy and medicine there were issues in drawing such conclusions or that data saturation had occurred. The idea of fully achieving data saturation may be unrealistic when applied to some populations or research questions; the resulting data may still contribute to the field and to further inquiry [34].

The purpose of conducting the research was to develop an intervention using a rigorous process of the constant comparative iterative method previously described and verification of findings with members of the discipline as well as formation of an audit trail. Researchers focused on ensuring contextual awareness and relevance for the development of the intervention to enhance credibility of the research findings guided by the planned action framework, KTA cycle.

#### Integration and application of the TDF

The integration phase of an exploratory sequential mixed methods study often leads to the development of an intervention or survey. In the process of building and connecting, the researcher has to decide or make key decisions about what aspects of the qualitative findings to build on in the development of the quantitative entity [11]. In our study, the qualitative findings (themes) were utilised and researchers determined what specific barriers, facilitators, and strategies from phase 1 would need to be addressed in the development of CASP. We utilised the TDF that focuses on behaviour change and developed CASP to promote changing the behaviour of clinicians (NPs) to improve the uptake of evidence (C-CHANGE guideline) in clinical practice.

**Fig. 1:**
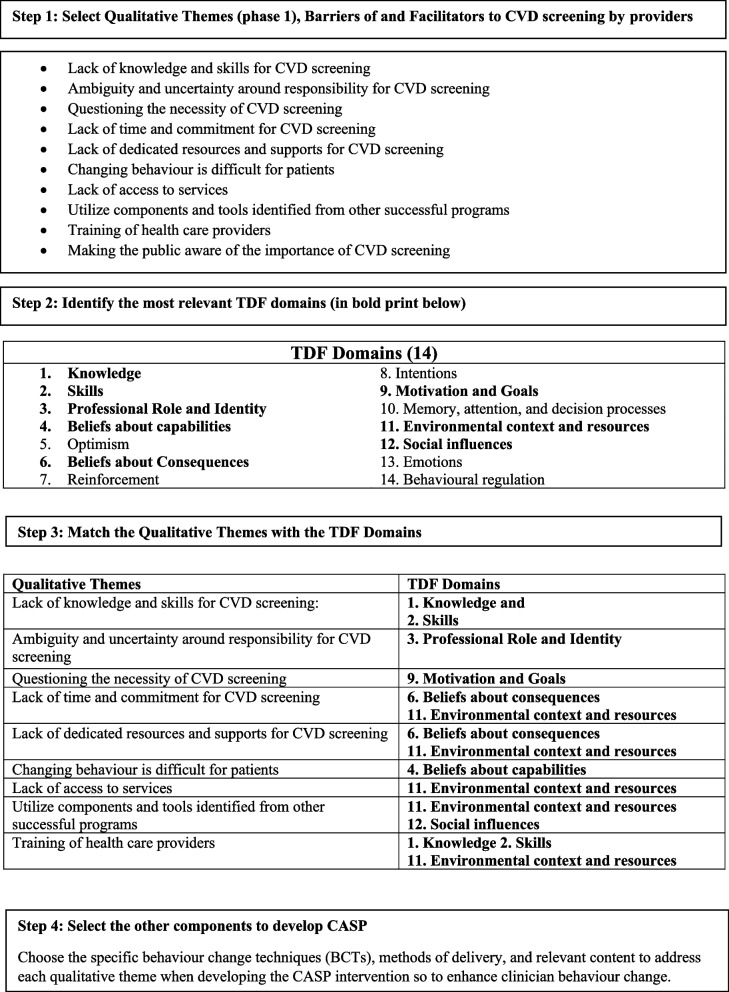
Integration: Qualitative Themes and the Application of the TDF, BCTs, and other Components

To develop CASP, the theory-informed intervention, the researchers integrated findings from the focus group and interviews with selected TDF domains, behaviour change techniques (BCTs), and modes of delivery available in the local context. In Table 1, the integration is outlined in Steps 3 and 4. Researchers applied the TDF by selecting the theoretical domains relevant to the development of CASP See Fig. 1. Integration: Qualitative Themes and the Application of the TDF, BCTs, and other Components. Further details of the integration process are described in the section Development of the CASP Intervention.

## Results

### Findings from focus groups and interviews

It was important to explore the barriers to the uptake of CVD screening by HCPs and to consider potential facilitators and strategies within the local context to enhance behaviour change of clinicians. The knowledge gained during the individual interviews and focus groups was used to build the components of the CASP intervention. While there were other qualitative results that could be reported, the researchers focused on selecting the more relevant findings to report in this manuscript. The qualitative findings chosen for this manuscript were the primary drivers for the intervention development. By identifying and presenting the HCPs’ barriers and facilitators to CVD screening, we could demonstrate, for example, how the components of our intervention addressed the specific barriers and promoted change in clinician behaviour. Even though the patients’ perspectives were not highlighted or obvious in this manuscript, their perspectives were taken into consideration in the development of intervention components to encourage participation in CVD screening.

### Barriers to CVD screening

There were seven (7) themes associated with the barriers to CVD screening: *1) lack of knowledge and skills for comprehensive CVD screening using the C-CHANGE Guideline; 2) ambiguity and uncertainty around responsibility for CVD screening; 3) questioning the necessity of screening in light of the Choosing Wisely Campaign; 4) lack of time and commitment for CVD screening; 5) lack of dedicated resources and organizational supports for CVD screening; 6) changing behaviour is difficult for patients; and, 7) lack of access to health services (within the local context).*

1. One theme that emerged from data analysis was the *lack of knowledge and skills for comprehensive* CVD screening using the C-CHANGE guideline. NPs often screen for individual CVD risk factors but were unfamiliar with the C-CHANGE guideline as it may not be widely disseminated in clinical practice. When NPs were questioned about their knowledge of the C-CHANGE guideline, they had not heard of them. One NP commented: "No I have never heard of it (the C-CHANGE Guideline)." Another NP responded: "….so they really just put it all together from all the guidelines that are out there?"

Other health professionals and managers were also not familiar with the existence of the C-CHANGE guideline when questioned during the individual interviews.

2) The second theme *ambiguity and uncertainty around the responsibility for CVD screening* emerged from the NP focus groups. NPs recognized that comprehensive screening for CVD was important but were uncertain about whether they were fully responsible. Some NPs thought that family physicians were responsible; however, NPs recognized that they too had a role along with other providers such as dietitians, public health nurses, community health nurses, and diabetes educators. One NP stated: “The family physician is probably an obvious answer. But you know some people are connected with diabetic teams of dieticians and diabetic RNs…people are sometimes identified through that avenue.”

The interviews with the public health nurse and two dietitians suggested that they have a role in assessing for CVD risk factors or encouraging patients to change behaviour, but these health professionals agreed that screening was not part of their mandate and the major responsibility should fall to physicians, primarily, and NPs.

3) The third theme was *questioning the necessity of CVD screening in light of the Choosing Wisely Campaign*, a campaign that discourages family physicians and other practitioners from ordering unnecessary tests and procedures. Because of this campaign, some NP participants had begun to question whether certain screening tests, including CVD screening, should be done at all. One NP said, "…and provincially you're getting into the whole financial discussion now of the Choose Wisely Campaign about unnecessary diagnostic testing and everything so it's a delicate balance. It's a very individual decision as a practitioner."
4) Another theme was *the lack of time and commitment for CVD screening* in the current provincial health care climate of fiscal restraints. NPs recognized that implementing a new CVD screening initiative would take extra time in their daily routine. Getting extra resources such as staff to assist in this screening process was very unlikely because of the present climate of decreased resources. The pharmacist and physician interviewed were aware of the time and commitment required for implementing a comprehensive CVD screening program in the current fee-for-service system in this province.
5) Likewise, *lack of dedicated resources and organizational supports for CVD screening* was a similar theme that emerged through the interviews with the healthcare managers. There was no organizational priority for prevention nor resources allocated for implementation of a CVD intervention focused on prevention. The health managers were concerned about costs such as time and money associated with the implementation of a comprehensive CVD screening program. In addition, there was a question about the sustainability of such a program in light of the associated costs and necessary resources.
6) Another theme was *changing behaviour is difficult for patients*. The NPs were frustrated due to lack of interest by many patients to change unhealthy behaviours. NPs questioned the value and the purpose of screening when the patients are reluctant to participate in the screening process and change their behaviour. One NP commented: “What if the patient doesn’t want to take meds, doesn’t want to make changes…why do it, put them through it? So, the risk associated with pricking them and then the cost of it and…they’re not going to change things?” Another NP stated: “People are resistant to change, just generally speaking, not just about cardiac but I mean diabetes, everything…(it’s) the same thing.”

Dietitians also spoke about the fact that behaviour change is difficult. Changing behaviours associated with eating is difficult based on their experience with counselling patients with diabetes. One dietitian stated: “…patients don’t engage for the follow-up piece. They believe that it is a quick fix. They believe that you’re going to tell them what to do.” One dietitian reported that patients often say, “Tell me what to eat…they find it hard to get here.” During one of the focus groups with the public the following comment was made about the reality of not being able to successfully change behaviour: “I think sometimes doctors…asking someone to change their diet, change their cholesterol level is not really effective. I think that’s the bottom line.”

7) Lastly, NPs noted that for patients in remote and rural areas there is a *lack of access to services*. One NP suggested that if people do not actually come to the clinic, then how would any program make a difference. “…Yeah, but it (the CVD screening program) only catches the ones who come to the clinic." Therefore, the people who would truly benefit from the program are not accessing preventative services.

For the urban group, wait times and lack of time given by family practice physicians to individualized care was discussed during the interview. “And it's not only that, people say oh go to my family doctor and that's a two-hour wait. I can't get in to see him for a month. How does that work?" Another commented: "Yeah, and then of course, you forget about it because I can't see my family doctor for two months or I can't get an appointment and then it's a five-hour wait." During focus groups interviews, members of the public in both urban and rural areas voiced their concern about their HCPs not taking time to focus on CVD screening.

### Facilitators for CVD screening

There were two facilitator themes for CVD screening: the first theme was: 1) knowing who to screen for CVD was obvious, but the timing of screening was not clear, and the second theme was 2) utilise components and tools identified from other successful provincial screening initiatives.

1. The first theme, *knowing who to screen for CVD was obvious, but the timing of screening was less clear*, captured the realization by NPs that there was a high prevalence of CVD risk factors in the province of NL. NPs in the focus groups presented a variety of responses such as the following: “Age and co-morbidities” “Family history”, “their lifestyles – if they’re smokers or inactive.” “Aboriginal descent- they are considered high risk… so we begin screening (earlier).” Another NP proclaimed: “I think just being a Newfoundlander is a risk factor!” The other health professional groups and managers recognized that there is a high prevalence of many CVD risk factors and conditions, so they believed CVD screening was obviously very important for all the people in NL.

NPs recognized that the ideal timing for CVD screening needed to be individualised given the many factors to be considered. NPs agreed that early screening is best especially for people in NL with a significant family history and given the fact that the province has the highest rates of CVD in Canada. Another NP stated: "… I think one standard age would be appropriate.” An NP explained the following: "…It depends – sometimes you get people on medication like in their 30s. Another NP stated the reality about the costs associated with screening: “I mean you’re not going to start screening everyone at 18…It’s expensive too, right.”

2) The second theme was *utilise components and tools identified from other successful provincial screening initiatives* could provide successful strategies for implementing CASP. In NL, the provincial cervical screening program and the Canadian Diabetes Strategy [35] initiative provide good examples of successful screening in the NL context. NPs concurred that considering the tools used for successful NL screening initiatives would be beneficial to determine strategies for CASP implementation. An NP commented about tools used during implementation of the Diabetes Strategy: “…. (using) the quick sheets like you got for diabetes… like I know with my diabetic patients I kind of just flip to the sheet…about what you do every 3 months, what you do every year. Like something like that – a quick sort of guideline thing.”

### Strategies for CVD screening

There were two themes identified around potential strategies that could be utilised for CVD screening.

1. One theme was the importance of *training of healthcare providers* such as NPs for the implementation of the CASP intervention to reduce the stress of fitting this program into daily clinical practice, and allaying fears of not knowing or understanding correct screening process according to the C-CHANGE guideline. Managers and the other health professionals agreed that training in comprehensive CVD screening was integral to success of the program. One HCP stated, “The important part here is they are quite familiar with the current guidelines and …they have to have a very good working knowledge of the tools like the Framingham, the heart score and whatever tools are available.” Having standardised training could ensure the consistent implementation of the intervention by HCPs.
2. The other theme from the focus groups and interviews with patients, HCPs, and managers was the importance of *making the public aware of the importance of CVD screening* so to address the challenge of patient engagement and participation in the CASP intervention. The need for a public awareness campaign and a champion to promote CVD screening with the public were viewed as vital to ensure success and sustainability of CASP.

### Development of the CASP intervention

We developed the CASP intervention by applying the TDF in the integration phase as described below. See Steps 3 and 4 in Table 1. The Table 2 contains a summary of the identified barriers, facilitators, and strategies from our qualitative study, the theoretical domains selected from the TDF, BCTs, modes of delivery, and the intervention components, including the content used.

**Table 2:** Application of the Theoretical Domains Framework for the Development of the Cardiovascular Assessment Screening Program (CASP)

**Barriers or Facilitators, or Strategies**	**Within which theoretical domain do the barriers, facilitators and strategies operate?**	**Which intervention components (behaviour change techniques (BCT) and modes of delivery (Mode) (Michie et al., 2013)** [14] **could overcome the modifiable barriers and enhance the facilitators and strategies to promote behavioural change associated with the content? (BCT, Mode, Content**^**a**^**)**
**Barrier** Lack of knowledge and skills for comprehensive CVD screening using the C-CHANGE guideline	**Knowledge**	**BCT:** Shaping knowledge; instruction on how to perform a behaviour
	**Skills**	**Mode:** Webinar, Online education module, CASP Website with online tools, resources, and C-CHANGE guidelines. Cardiovascular Access Database used as the online documentation system
		**Content:** Background information on CVD screening, and access to clinical practice guidelines (C-CHANGE). NP Toolkit (Provided resources and materials for NP behaviour change related to the screening process during intervention implementation)
**Barrier** Ambiguity and uncertainty around responsibility for CVD screening	**Professional role and identity**	**BCT:** Goals and planning-discrepancy between current behaviour and standard of practice
		**Mode:** Webinar; One-on-one facilitator support; Online education module
		**Content:** NP role in CVD screening, health promotion, adherence to clinical practice guidelines, and access to relevant nursing research. *ARNNL NP Standards of Practice* state the role of NPs to integrate health promotion at the individual and community level in clinical practice and research
**Barrier** Questioning the necessity of CVD screening in light of the Choosing Wisely Campaign	**Motivation and goals**	**BCT**: Goals and planning-Action planning (including implementation intentions)
		**Mode**: CASP materials were in congruence with the Choosing Wisely Campaign recommendations. Feedback questionnaires sent to NPs
		**Content**: CASP contained information that was in congruence with the Choosing Wisely recommendations
**Barrier** Lack of time and commitment for CVD screening	**Beliefs about consequence**	**BCT:** Commitment; Social support
		**Mode:** Email and phone calls; Webinar; Online access to facilitator and technical support during research study
	**Environmental context**	**Content** Streamlined the process of CVD screening, management, and documentation through online resources for easy access and to reduce time and costs associated with NP participation in screening process. Ongoing support from CASP facilitator and technical support during intervention implementation
**Barrier** Lack of dedicated resources and organizational supports for CVD screening	**Beliefs about consequences**	**BCT:** Commitment; Social support
		**Mode:** Email and phone calls; Webinar; Online access to facilitator and technical support during research study
	**Environmental context**	**Content** Streamlined the process of CVD screening, management, and documentation through online resources for easy access and to reduce costs associated with NP participation in screening process within organizations. Ongoing support from CASP facilitator and technical support during intervention implementation
**Barrier** Changing behaviour is difficult for patients	**Beliefs about capability**	**BCT**: Repetition and substitution-Behaviour rehearsal/practice
		**Mode**: Online educational module containing PowerPoint presentation
		**Content**: Focused on behaviour change of NPs and behaviour change of patients. Focused on the application of the Trans Theoretical Model and motivational interviewing techniques for NP behaviour change. Access to My Heart Healthy Plan focused on a patient-centred approach where the onus is on patient self-management and patient control of decision-making and goals for behaviour change in collaboration with the NP
**Barrier** Lack of access to services	**Environmental context**	**BCT**: Antecedents; restructuring the physical environment
		**Mode:** Online CASP website accessible to HCPs and separate access for members of the public
		**Content:** The CASP intervention and other resources for NPs and patients in urban and rural remote areas of NL. Resources to promote heart health for screening and management and self-management
**Facilitator** Utilize components and tools identified from other successful provincial screening programs	**Environmental context**	**BCT**: Goals and planning-problem-solving
	**Social influences**	**Mode**: Access to resources for providers and patients through the CASP Website, HCP Toolkit, online links to other resources
		**Content**: Use of the CASP resources such as Heart Health Pamphlet, diabetes quick sheets, patient education materials, and screening tools for NPs to use in daily practice
**Strategy** Training of health care providers for implementation of a comprehensive screening intervention to reduce stress of fitting this program into daily clinical practice	**Knowledge** **Skills** **Emotions** **Environmental context**	**BCT**: Shaping knowledge-instruction on how to perform a behaviour; Repetition and substitution behavioural rehearsal/practice; Social support emotional
		**Mode**: Webinars, online educational module, support from researchers through various means. Online support from CASP Website, online support through Educational module, online CVD Database
		**Content**: Introduction of the educational module and other resources to be used during CASP implementation. Educational module contained videos on correct technique on how to do skills correctly according to CPGs. Support available to NPs participating in by CASP facilitator through email, phone, or in-person during CASP study implementation and availability of online resources
**Strategy** Making the public aware of the importance of CVD screening	**Beliefs about consequences** **Environmental context**	**BCT**: Antecedents-restructuring the physical environment, changing exposure to the cues for the behaviour
		**Mode:** HCP Toolkit**,** Send materials to various RHAs, posters, pamphlets, media campaigns
		**Content**: Distribution of Heart Healthy Posters in regional health authorities across the province of NL. NPs advertising specific days for CVD screening

^a^*BCT* behaviour change technique chosen, *MODE* method of delivering. Content: information delivered

### Theoretical domains

In our research, eight of the 14 theoretical domains of the TDF were relevant to the development of the CASP screening intervention. Table 2 lists the eight domains and summarizes the application of the TDF for the development of CASP in columns one and two. To illustrate our application of the TDF for the development of the CASP, we have selected the identified barrier in the first column of Table 2, lack of knowledge and skills for comprehensive screening using the C-CHANGE guideline. This barrier operates within the two theoretical domains of Knowledge and Skills.

### Behaviour change techniques

Following the identification of the theoretical domains for the identified barriers and facilitators, the next step was to select the behaviour change techniques. These are summarized in column 3 of Table 2. The structural taxonomy of BCTs by Michie et al., 2013 [14] was used during this study as similarly described for other behaviour change interventions found in the literature [12, 18]. The BCTs are reproducible, observable actions that can support the behaviour change of individuals (or changes within organizations) [13]. In our example from Table 2, lack of knowledge and skills for comprehensive screening using C-CHANGE guidelines, the change technique that was selected from the BCT taxonomy was “shaping knowledge, instructions on how to perform a behaviour” (within the Knowledge and Skills domains) to overcome the barrier of lack of NP knowledge and to provide a pathway to changing behaviour. For example, shaping NP cognitive knowledge occurred through access to one of the CASP components (online education module) for specific instructions on how to perform physiological measurements accurately during CVD screening. In addition, the NPs learned how to measure waist circumference accurately by viewing a colourful illustration provided in the CASP education module that contained detailed instructions on how to perform this behaviour.

### Modes of delivery and content

The modes of delivery for the intervention were determined in terms of the feasibility, practicality, and resources within the local context. The methods or modes of delivery as described by Michie et al. 2013 are procedures for the delivery of the content of the intervention [14]. These are also summarized in column three of Table 2. Modes of delivery such as webinars, CASP website, and online resources were intended to provide information that could potentially encourage clinician behaviour change. In our example from above in Table 2, the modes of delivery for the intervention content to enhance NP knowledge and skills were the following: a webinar, an online education module, the CASP website, and other online resources.

Table 2 also provides examples of the content of the CASP intervention to be delivered. In our example from above, the content to be covered to enhance the knowledge and skills of the NPs included background information on CVD screening and access to an interactive decision tool based on the C-CHANGE guideline. Other content contained in CASP was a HCP Toolkit and resources for NPs to use during the screening process. This intervention content addressed the identified barrier of NP lack of knowledge and skills for comprehensive screening.

We have illustrated one example from Table 2; a similar process was followed for all of the domains. Even though the modes of delivery were similar, the content is very specific to address each identified barrier, facilitator, or strategy.

### Overview of the developed CASP intervention

**Fig.2:**
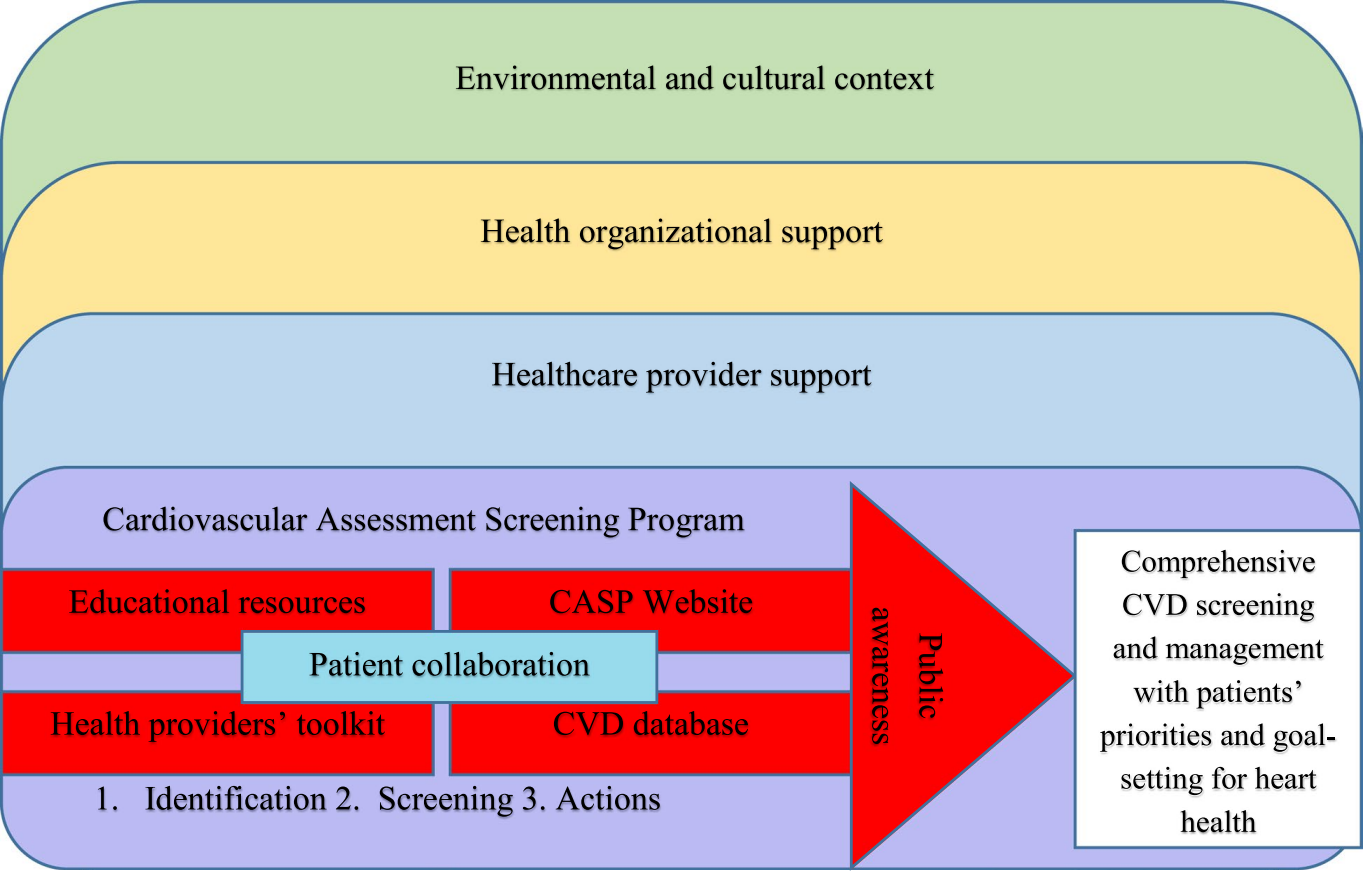
The Cardiovascular Assessment Screening Program (CASP)

The CASP intervention developed consisted of four main components: 1) CVD database, 2) CASP website, 3) HCP toolkit, and 4) an online educational module [17], see Fig. 2. The Cardiovascular Assessment Screening Program (CASP). The CVD screening process by HCPs, with patient collaboration throughout, occurs with support from health care organisations and the broader environmental context.

The CVD database was set up as a worksheet to guide NPs through CVD screening while also providing a tool for documentation. CVD risk factors, physiological measurements, current medications, Framingham Risk Score, Heart Age, heart health priorities, and the individual participants’ personalised goals. The other CASP components were also easily accessible online in daily clinical practice through a website, toolkit, and educational module. The CASP website, accessible for HCPs and patients, contained background information about the research study and tools for accurate measurement to ensure consistent data collection. CVD screening and recommendations for management of the risk factors were based on the C-CHANGE guideline embedded in the CASP website intervention previously described [17].

## Discussion

The aim of this study was to address a gap in the literature related to HCPs’ lack of uptake of CVD screening and management using current CPGs. To address this gap, we used KT and a behaviour change theory in our research study. Firstly, we used the planned action framework, KTA cycle, with guideline adaption [10]. This framework guided this research to determine effective strategies for knowledge translation of the C-CHANGE guideline into daily clinical practice in NL. The first phase of the KTA Framework involved identifying the C-CHANGE guideline as the expert knowledge; the second phase required identifying the barriers and facilitators to knowledge use and tailoring an intervention to be relevant to the NL context. Researchers consulted with key target groups to determine the barriers to and facilitators of implementing CASP within the local context. During the qualitative study, individual interviews and focus groups held with a variety of participant groups, not just NPs, but also clinicians, managers, and the public, ensured that many different perspectives informed the provisional CVD screening program, CASP, making it relevant to the local practice and health system prior to implementation. The process of development of the CASP intervention adds to implementation science literature through knowledge translation of evidence into practice in terms of illustrating operationalisation of research evidence from the C-CHANGE guideline into clinical practice.

Secondly, in our study, we used the TDF to promote clinician behaviour change to improve uptake of CPGs in daily practice. Other researchers have followed the process of applying behaviour change theories such as the TDF, but our research study adds to the literature by focusing on CVD screening and management. While other researchers have developed interventions for screening for CVD risk factors, they often focused on screening single risk factors or two to three factors; in contrast, CASP utilises a comprehensive systematic approach to identify and manage multiple risk factors simultaneously. As well, utilising the TDF promoted a broader range of strategies to address multiple issues. For example, by applying the TDF and considering the theoretical domain ‘environmental context’, researchers thought more broadly about the issue of lack of dedicated resources and organizational supports for HCPs. Thus, by creating online resources such as the electronic CVD database and the CASP website embedded with CPGs, HCPs had easy and timely access so to streamline the process of CVD screening, management, and documentation. Without using the TDF, it would have been more difficult for researchers to conceptualise or consider improving access to the CPGs for HCPs’ use for CVD screening and management in the broader provincial environmental context.

The next phase of this mixed methods study focused on testing the CASP intervention with NPs and patients in community settings across NL, Canada, to determine the effectiveness of the CASP in comprehensive CVD screening and management of patients aged 40–74 years without established CVD or vascular disease. In the final integration phase, the findings from both the qualitative and quantitative studies further refined the CASP intervention [17].

### Strengths and limitations

Strengths of this research study are in the use of mixed methods research design and a theoretical foundation leading to the development of a novel intervention for CVD screening and management. We have developed a new comprehensive theory-informed intervention for CVD screening and management but other researchers may choose to use a similar process for development for other CPGs to address other health conditions. Use of a strong systematic comprehensive approach and CASP may be useful to customise or to adapt for different conditions and populations e.g., assessing CVD risk for individuals within vulnerable populations such as those with severe mental illness at significant CVD or metabolic risk and poorer health outcomes.

Our study has several limitations; qualitative research that identified barriers and facilitators was based on a small sample of HCPs, patients, and administrative personnel due to difficulties in recruiting participants and there were time constraints of the dissertation research. Use of the TDF, in this study, while providing a framework and theoretical basis for the intervention development to address behaviour change of clinicians, may have limitations related to the selection of specific domains and determining at what level to apply such constructs, which tends to be time-consuming and resource intensive. The research occurred in one eastern province with a small population base so might not be generalisable to the wider Canadian population or beyond. In spite of these limitations, it is still worthy of testing the intervention to determine its effectiveness with individuals at risk and health provider participants.

## Conclusion

We used a systematic comprehensive theory and evidence-informed process for KT, which may be useful for other researchers in the future studies. This article adds to the literature to illustrate the process of integrating qualitative study findings with the TDF to develop an intervention for CVD screening and management.

## Supplementary Information

**Additional file 1.** Phase 1 participants.

**Additional file 2.** Interview guides for focus group & individual interviews.

## Data Availability

The datasets generated and analysed during the current study are not publicly available due to the potential of identifying individual participants but are available from the corresponding author on reasonable request.

## Abbreviations

BCT: Behaviour Change Techniques
CASP: Cardiovascular Assessment Screening Program
C-CHANGE: Canadian Cardiovascular Harmonized National Guideline Endeavour
CPG: Clinical Practice Guideline
CVD: Cardiovascular Disease
HCP: Healthcare Provider
NP: Nurse Practitioner
PI: Principal Investigator
PHAC: Public Health Agency of Canada
TDF: Theoretical Domains Framework
WHO: World Health Organization
