# Supplementary material for: Development of the Cardiovascular Assessment Screening Program (CASP) using the qualitative findings of a mixed methods study and applying the TDF to address the barriers of and facilitators to comprehensive screening for cardiovascular disease
Source: BMC Prim Care. 2023 Mar 7;24:65. doi: 10.1186/s12875-023-02022-8 (PMC9990229; doi:10.1186/s12875-023-02022-8)
Supplement: Supplementary file 1 — Additional file 1. Phase 1 participants. [file 12875_2023_2022_MOESM1_ESM.docx]

**BMC Supplementary File**

**Additional file 2**

**Interview Guides for Focus Group & Individual Interviews**

**Focus Group Questions**

**(Members of the health professional groups)**

**Opening script:** *Thank you for agreeing to participate in this [focus group/interview] today. We really appreciate your time and interest in the topic of screening for heart disease in NL. If you haven’t given me your signed consent form, you can give it to me now. We do ask that you keep our discussion confidential so please do not to discuss what we have talked about with friends, family, or coworkers outside this room. You do not have to respond to any questions that you are not comfortable to answer. Your comments will not be linked to in any way to your name or no one will be able to identify you in any way. Thank you again for your participation. So, today’s discussion is about ways to increase screening for cardiovascular disease in NL. There are three main steps in the screening process. We are going to talk about each of these and then have a general discussion. Step 1 is identifying patients to screen. Step 2 is actually carrying out the screening process. Step 3 is about acting on the screening test results.*

[**Prompt:** Step 1. Identification of patients to screen]

*First, we are going to talk about ways that we can identify people who need to be screened.*

1. Do you routinely screen patients for CVD? If no, whose responsibility is it? What is the best way to identify people to screen for CVD?
2. How do you usually identify people to screen for CVD?
   1. **Prompt:** Do you review patient’s charts, current patient rosters, or do you have clinical reminder systems (electronic or paper-based) that flag charts according to the patient’s age? Do you receive referrals from other professionals to screen patients?
   2. What works for you?
   3. Does it work well?
   4. What are some issues related to screening for CVD?
   5. Are there lessons to be learned from screening for other conditions?
3. Is knowing who to screen a problem?
   1. When do you specifically screen men? Women? Children?
4. What kind of organizational supports would help you with screening patients for CVD? (For example: more time, EHR, incentives, policy to make it a priority, etc).

**[Prompt:** Step 2. Screening patients]

*Now we are going to talk about actually screening the patients by taking measurements (such as measuring waist circumference, calculating BMI or taking a blood pressure) or using other tools to screen (such as CPGs, Framingham global risk assessment) and discuss some of the barriers or issues that may be occurring. Now let’s talk about screening.*

1. We have a preliminary program developed based on the C-CHANGE guidelines. Would you be comfortable with using C-CHANGE? What kind of access do you need to find the C-CHANGE guidelines? Would this table help you? [show C-CHANGE table]
2. What CPGs do you use for screening adults in your practice? What guidelines would be useful to access to help you know what to screen for and when?

*We also suggest that you use a global CV risk tool*

1. What tools (global risk assessments) do you use to screen for CVD risk factors in your clinical practice?
   1. Is there any concerns with using the Framingham Risk Tool?
   2. Do you use another tool?
   3. How do you use this tool?
   4. Would you be comfortable to change your approach?
   5. Do you use the online calculator?
   6. Do you calculate the “Heart Score”?

*Part of the screening process involves doing physical measurements*

1. What physical measurements do you perform when screening patients?
   1. Body mass index
   2. Weight measurement
   3. Height measurement
   4. Waist circumference measurement
   5. Blood pressure measurement
   6. What do you use as measurement tools?
   7. Do you take blood samples in the clinic?
   8. Do *you* normally take these physical measurements?
   9. Are there barriers or issues associated with taking physical measurements?
2. Would you use the proposed screening intervention in your clinical practice? Why or why not? (Show one page explanation of proposed screening intervention).
   1. What would you need to convince you or to help you implement this screening initiative?
   2. What suggestions do you have to improve this proposed screening intervention?
3. Is it easy to get participants to engage in a screening intervention for CVD?

[**Prompt:** Step 3. Acting on the results of screening]

*Next, we want to know what sort of actions are required after completing the screening process.*

1. Where do you document your findings related to screening patients?
   1. Do you use the electronic health record (EHR)?
   2. Does the EHR work for you?
   3. Is it easy to find information for follow-up after screening patients?
   4. What system do you use?
2. After screening patients, how do you follow-up on the results of testing and screening? What do you need to be able to follow-up on screening results (tools and resources)? Do you set up appointments or phone patients when results come back? Do you send referrals to other practitioners? Do you do patient education yourself?

*Part of following up on screening results is asking patients to change behaviour or to follow advice.*

1. Are you familiar with motivational interviewing? Behavioural change counselling (Ask, Advise, Assist)? How do you ensure that you are providing patient-centred care?
2. Do you have any suggestions for patient engagement for following through with your suggestions after being screened? Is there something that needs to be included

in the preliminary program to help with patient engagement?

**[Prompt:** General questions about screening]

*Now we would like your opinion on some more general questions related to screening.*

1. What are some barriers to implementing a screening intervention? What suggestions do you have to address them?
2. What are some facilitators to implementing a screening intervention?
3. Are there any other strategies to increase CVD screening?
4. How important would training be for this intervention? Would you want to have tools or would you like to have training related to this screening intervention?
5. Do you have any suggestions for screening patients based on your own experience?

**Focus Group Questions**

**(Members of the general public)**

**Opening script:** *Thank you for agreeing to participate in this [focus group/interview] today. We really appreciate your time and interest in the topic of screening for heart disease in NL. If you haven’t given me your signed consent form, you can give it to me now. We do ask that you keep our discussion confidential so please do not to discuss what we have talked about with friends, family, or coworkers outside this room. You do not have to respond to any questions that you are not comfortable to answer. Your comments will not be linked to in any way to your name or no one will be able to identify you in any way.*

**[Prompt:** Identification of patients to be screened]

*First of all, we would like your advice on how to best contact you to get you involved in screening for heart disease.*

1. Would you like to participate in a screening program to assess your risk for heart disease or stroke? Why or why not?
2. What is the best way to get you involved in screening? Invitations to be sent via regular mail, email, or a telephone call from your HCP?
3. When do you think it is the best time for you to be screened for heart disease? What age?

[**Prompt:** Screening process]

*Now, we are going to talk about the screening process itself and what that means.*

1. What concerns do you have about the actual screening tests (such as getting your BP taken, physical measurements like your height, weight, or having a blood test done)?
2. Are you interested in knowing about your overall risk for developing heart disease?
3. What are some barriers (or things that make it difficult) to participate in a screening program?
4. What are the facilitators (or things that make it easier) to participate in a screening program?
5. What can the health care provider do to address the concerns and barriers that you have identified?

**[Prompt:** Acting on screening test results]

*After the screening tests are completed, your health care provider wants to share the results of these tests or measurements with you and make some recommendations.*

1. Would you follow recommendations (or advice) from a health care provider that could possibly reduce your risk of developing CVD in the future?
2. What makes it difficult to follow the advice given to you by a health care provider?
3. What makes it easier to follow the advice given to you by a health care provider?
   1. Would printed materials be helpful?
   2. Website resources?
   3. Would a dietician be helpful to make changes to your diet?
   4. Counselling by your health care provider?
   5. Group support to make necessary changes?

*A screening program is being developed and we need your advice on whether or not you think it will work or what changes should be made before using it*

*.*

**[Prompt:** Proposed screening intervention]

1. Would you participate in this screening program? Why or why not?
2. What would you change about this screening intervention?

**Interview Questions**

**(Managers)**

**Opening script:** *Thank you for agreeing to participate in this interview today. We really appreciate your time and interest in the topic of screening for heart disease in NL. If you haven’t given me your signed consent form, you can give it to me now. We do ask that you keep our discussion confidential so please do not to discuss what we have talked about with friends, family, or coworkers outside this room. Thank you again for your participation today. So, today’s topic is about ways to increase screening for heart disease in NL.*

**[Prompt:** Support for health professionals to do screening for CVD]

*First of all, we would like your opinion on what can be done to support NPs or others to screen for CVD in their clinical practice.*

1. What are your thoughts about NPs doing systematic CVD screening? Why?
2. If it can be supported, what can be done from an organizational support point of view?
   1. Prompt: Organizational support according to the literature means providing time, resources, EHR, relief from other responsibilities, etc.
   2. What needs to be done to ensure that it can be implemented in terms of other responsibilities currently performed by HCPs?

[**Prompt:** Proposed screening intervention]

*Now, we would like your opinion on a provisional or tentative screening initiative that has been developed. These are the elements of the program and how they are related.* (Show one page of proposed screening intervention and explain it).

1. Are you willing to support this provisional CVD screening intervention and encourage NPs to implement it? Why or why not? Do you think that it would be useful for other HCPs?
2. From your perspective, what suggestions do you have for improvements or effective strategies for the successful implementation of this screening intervention?
3. What kind of supports can you provide for an initiative such this screening intervention?
