# Supplementary material for: Development of the Cardiovascular Assessment Screening Program (CASP) using the qualitative findings of a mixed methods study and applying the TDF to address the barriers of and facilitators to comprehensive screening for cardiovascular disease
Source: BMC Prim Care. 2023 Mar 7;24:65. doi: 10.1186/s12875-023-02022-8 (PMC9990229; doi:10.1186/s12875-023-02022-8)
Supplement: Supplementary file 2 — Additional file 2. Interview guides for focus group & individual interviews. [file 12875_2023_2022_MOESM2_ESM.docx]

**BMC Supplementary File**

**Additional file 1**

**Phase 1 Participants**

| **Target groups (Roles)** | **Urban** | **Sex M/F** | **Rural** | **Sex M/F** | **Total** |
| --- | --- | --- | --- | --- | --- |
| **Focus Groups** | **(n=number of participants)** |  | **(n=number of participants)** |  | **N=Total participants** |
| **Nurse Practitioners**  **(3 focus groups)** | **1 (n=2)** | **Females (2)** | **1 (n=4)** | **Males (1)**  **Females (3)** |  |
|  | **1 (n=3)** | **Males (1)**  **Females (2)** |  |  |  |
|  |  |  |  |  |  |
| **General Public**  **(2 focus groups)** | **1 (n=6)** | **Males (1)**  **Females (5)** | **1 (n=5)** | **Females (5)** |  |
| **Individual Interviews** | **Urban** |  | **Rural** |  |  |
| **Nurse Practitioners** |  |  | **n=1** | **Females (1)** |  |
| **Public Health Nurses** | **n=1** | **Females (1)** |  |  |  |
| **Physicians** | **n=1** | **Males (1)** |  |  |  |
| **Dietitians** | **n=2** | **Females (2)** |  |  |  |
| **Pharmacists** | **n=1** | **Males (1)** |  |  |  |
| **Managers** | **n=3** | **Females (3)** |  |  |  |
| **General public** | **n=1** | **Males (1)** |  |  |  |
|  |  |  |  |  |  |
| **Focus group and individual interview**  **Participants** | **n=20** | **Males (5)**  **Females (15)** | **n=10** | **Males (1)**  **Females (9)** | **N=30**  **Males (6)**  **Females (24)** |
